# Differential MHC class I expression in distinct leukocyte subsets

**DOI:** 10.1186/1471-2172-12-39

**Published:** 2011-07-15

**Authors:** Justin M Greene, Roger W Wiseman, Simon M Lank, Benjamin N Bimber, Julie A Karl, Benjamin J Burwitz, Jennifer J Lhost, Oriana E Hawkins, Kevin J Kunstman, Karl W Broman, Steven M Wolinsky, William H Hildebrand, David H O'Connor

**Affiliations:** 1 Department of Pathology and Laboratory Medicine, University of Wisconsin-Madison, Madison, 53706, Wisconsin, USA; 2 Wisconsin National Primate Research Center, University of Wisconsin-Madison, Madison, 53715, Wisconsin, USA; 3 Department of Microbiology and Immunology, University of Oklahoma Health Sciences Center, Oklahoma City, 73104, Oklahoma, USA; 4 Division of Infectious Diseases, Northwestern University Feinberg School of Medicine, Chicago, 60611-2826, Illinois, USA; 5 Department of Biostatistics and Medical Informatics, University of Wisconsin-Madison, 53715, Wisconsin, USA

## Abstract

**Background:**

MHC class I proteins are partly responsible for shaping the magnitude and focus of the adaptive cellular immune response. In humans, conventional wisdom suggests that the HLA-A, -B, and -C alleles are equally expressed on the majority of cell types. While we currently have a thorough understanding of how total MHC class I expression varies in different tissues, it has been difficult to examine expression of single MHC class I alleles due to the homogeneity of MHC class I sequences. It is unclear how cDNA species are expressed in distinct cell subsets in humans and particularly in macaques which transcribe upwards of 20 distinct MHC class I alleles at variable levels.

**Results:**

We examined MHC gene expression in human and macaque leukocyte subsets. In humans, while we detected overall differences in locus transcription, we found that transcription of MHC class I genes was consistent across the leukocyte subsets we studied with only small differences detected. In contrast, transcription of certain MHC cDNA species in macaques varied dramatically by up to 45% between different subsets. Although the Mafa-B*134:02 RNA is virtually undetectable in CD4+ T cells, it represents over 45% of class I transcripts in CD14+ monocytes. We observed parallel MHC transcription differences in rhesus macaques. Finally, we analyzed expression of select MHC proteins at the cell surface using fluorescent peptides. This technique confirmed results from the transcriptional analysis and demonstrated that other MHC proteins, known to restrict SIV-specific responses, are also differentially expressed among distinct leukocyte subsets.

**Conclusions:**

We assessed MHC class I transcription and expression in human and macaque leukocyte subsets. Until now, it has been difficult to examine MHC class I allele expression due to the similarity of MHC class I sequences. Using two novel techniques we showed that expression varies among distinct leukocyte subsets of macaques but does not vary dramatically in the human cell subsets we examined. These findings suggest pathogen tropism may have a profound impact on the shape and focus of the MHC class I restricted CD8+ T cell response in macaques.

## Background

MHC class I genes are critical to the development of the cellular immune response. The products of these genes are cell surface glycoproteins expressed on nearly every nucleated cell. These molecules present short fragments of endogenous proteins to surveillance CD8+ T cells. Once a cell becomes cancerous or is infiltrated by an intracellular pathogen, MHC class I proteins present these foreign peptide fragments to CD8+ T cells. CD8+ T cells can secrete cytokines and kill cells presenting specific MHC-antigen complexes, preventing the spread of a pathogen or tumor development. Both intracellular pathogens and tumors subvert CD8+ T cell killing by altering MHC class I presentation. Decreasing surface expression of MHC class I proteins renders infected cells less visible to CD8+ T cells, allowing pathogens and tumors to survive and replicate undetected. Thus, developing a clear picture of MHC expression on the cell surface is a critical component of understanding the body's response to cancer and infection.

The classical human MHC class I loci are termed HLA-A, -B and -C. In contrast to the HLA, macaque MHC class I genes have experienced multiple gene duplications and deletions. Although macaques lack a homologue of the HLA-C locus, they have an expanded number of MHC class IA and IB loci encoding up to 19 distinct class I transcripts on a single haplotype [1-4]. Like humans, specific macaque MHC alleles have been associated with both susceptibility and resistance to disease [3].

The repertoire of MHC alleles and the level of expression of each of these alleles is a critical aspect of how the immune system responds to pathogens. HIV and other intracellular pathogens are known to preferentially infect distinct leukocyte subsets, thus the particular MHC class I alleles expressed by infected cells may define the repertoire of immune responses generated by an individual [5-7]. Additionally, it was recently demonstrated that MHC class I proteins can act as virus entry receptors [8]. In this circumstance, MHC expression may help define the tropism of a pathogen. Finally, the level of MHC expression on the cell surface is also critical to natural killer cell signaling where MHC molecules can act as activating or inhibitory ligands for natural killer cells [9]. Basal expression levels of certain MHC may determine how the body responds to pathogens that subvert MHC presentation. These facts indicate that a thorough examination of MHC expression is critical to understanding the body's susceptibility and response to pathogen [10]. Furthermore, MHC class I transcript expression in macaques is particularly interesting considering the large number of potential transcripts being expressed by a single cell.

It is difficult to reconcile macaque expression of more than three-dozen distinct MHC class I sequences provided our current understanding of cellular immunity. Researchers classically view expression of MHC as a balance between having sufficient alleles to generate a diversity of responses, and having too many alleles which may result in negative thymic selection of otherwise effective and important responses [1]. It remains unclear how macaques maintain expression of, at least, 10 MHC class I alleles while sustaining a functional and diverse CD8+ T cell response. Recent studies from the macaque revealed that MHC class I molecules expressed at low levels in the macaque PBMC do not appear to contribute to the restriction of SIV-specific CD8+ T cell responses [11]. These results demonstrate that we do not understand the function of the alleles expressed at lower levels or that we have simply not identified the tissues in which these alleles are expressed at higher levels.

While much is known about total MHC class I expression on individual cells, quantifying the contribution of individual alleles to overall expression is much more difficult. Thus far, almost all expression studies have relied upon using artificial expression of MHC class I alleles in suitable MHC null cell lines and pan-MHC class I antibodies [12-15]. The recent introduction of quantitative real-time PCR assays that make it possible to compare HLA-A, -B, and -C locus-specific transcription in normal human tissues have provided at least preliminary evidence for preferential transcription of the HLA-B locus in some individuals' PBMC [16]. Alternatively, researchers have used immortalized cell lines to study MHC class I expression. These studies are removed from the *in vivo *environment and rely on oversimplification of the natural cellular MHC class I milieu. Furthermore, immortalization may depend on techniques, like viral infection, that alter MHC class I expression. It was previously demonstrated in mice that the presence or absence of certain alleles can affect the surface expression of alleles on the opposite haplotype and ultimately change the pattern of cellular immune responses generated by an individual [17]. This study suggests that characterizing a given MHC class I allele in isolation will, at best, provide an incomplete picture of expression. More recently investigators have used locus specific antibodies to interrogate HLA-C expression in HIV infected individuals [18]. Thomas *et al*. found that individuals with highly expressed HLA-C alleles maintained lower chronic phase HIV viral loads. These results indicate that differences in MHC expression can be correlated with control of viral replication and disease pathogenesis.

Previous studies have not examined how expression of MHC class I alleles varies among cell types such as individual leukocyte subsets *ex vivo*. We examined expression of MHC in both human and macaque leukocyte subsets. Mauritian cynomolgus macaques (MCM) have limited MHC diversity providing an opportunity to study animals that are homozygous or heterozygous within the MHC region [19-23]. Here we use high-throughput pyrosequencing of a variable region of the MHC class I sequence to assess relative transcript abundance of specific alleles in purified leukocyte subsets [22]. We did not observe large differences in MHC class I transcription among the leukocyte subsets in the humans that we examined. In contrast, we found that transcription varied for three MCM MHC class I genes, with one cDNA species differing as much as 45% among distinct leukocyte subsets. We also identified differentially expressed transcripts in the rhesus macaque that varied up to 15% among different leukocyte subsets. Moreover, we observed that this variability in transcript number equated to differences in protein expression of several alleles including those that restrict SIV-specific CD8+ T cell responses. To our knowledge, this is the first example of differential expression of classical MHC class I transcripts by distinct, surface marker defined, normal primate cells.

## Methods

### Subjects

Two HIV-negative subjects were enrolled in Madison through the University of Wisconsin Hospital. This study was approved by the Wisconsin Health Sciences Institutional Review Board.

### Animal Care and Use

**Figure 1:**
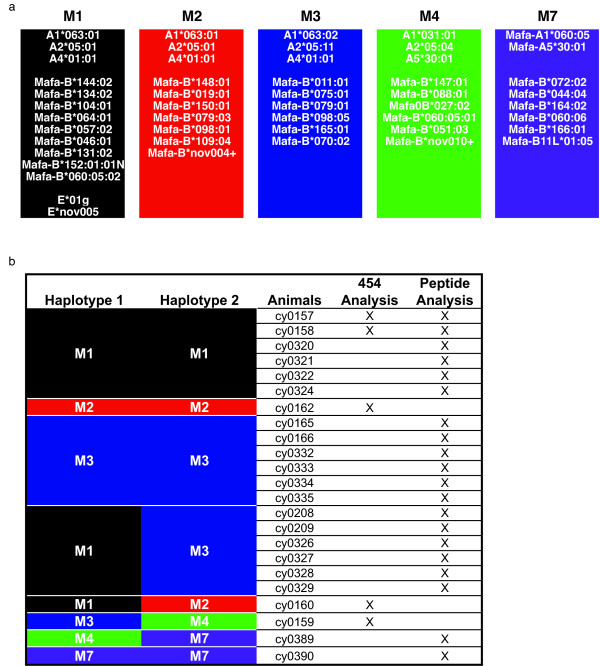
**Cynomolgus macaques used in the study**. a) Each column represents a different haplotype with previously defined MHC class I alleles. b) Animals used throughout the study. Haplotypes are indicated to the left of the animal name. An × is marked in columns to indicate whether the animal was used in the 454 analysis, fluorescent peptide analysis or both.

Animals were cared for by the Wisconsin National Primate Research Center (WNPRC) according to protocols approved by the University of Wisconsin Research Animal Resources Center review committee. A table of the animals used in this study can be found in Figure 1. Sequencing analysis of MCM was performed prior to animal infections. Rhesus animals r03047, and r98030 were SIV positive while animals r02033 and rh2349 was uninfected at the time of this study. Protein expression studies were performed after animals were SIV+ (Figure 1). We used a total of twenty-three Mauritian cynomolgus macaques and four rhesus macaques.

### Microsatellite Typing of Mauritian Cynomolgus Macaques

Animals were haplotyped using a previously described panel of microsatellite markers spanning the ~5 Mb genomic MHC region [23]. These haplotypes were used to infer the MHC genotypes of the animals used in this study.

### Cell Lines

Transfectant cell lines were created as previously described [19]. Cells were maintained in complete media. Transfectants were maintained under G418 (Mediatech, Manassas, VA) drug selection.

### Cell Separations

Blood was drawn from macaques or human subjects into EDTA tubes at the WNPRC or University of Wisconsin Hospital respectively and layered over Ficoll-Paque Plus (GE Healthcare Bioscience, Uppsala, Sweden); peripheral blood mononuclear cells (PBMC) were isolated by density centrifugation. We then split the PBMC into several equal aliquots for each leukocyte separation. CD8+ T cells were isolated using an anti-CD8ß-phycoerythrin (PE) monoclonal antibody (Beckman Coulter, Fullerton, CA) and anti-PE beads with LS columns (Miltenyi, Auburn, CA). CD4, CD14, CD16, and CD20 cell separations used the single-step nonhuman primate or human kits available from Miltenyi. As each leukocyte subset was isolated, we froze cells in RNAlater (Ambion, Foster City, CA) and set aside aliquots of each sample for staining of cell surface markers. Each sample was stained with CD14 Fluorescein isothiocyanate (FITC) (BD Biosciences, San Jose, CA), CD4 phycoerythrin Cy5 (PE Cy5), CD19 phycoerythrin Cy7 (PE Cy7) or CD3 PE Cy7; CD8 Pacific Blue, CD20 allophycocyanin (APC), W6/32 APC or CD4 APC; CD3 Alexa Fluor 700 (A700) or CD14 A700 and CD8B PE (Beckman Coulter, Fullerton, CA), CD16 PE or CD20 PE and run on an LSRII flow cytometer (BD Biosciences, San Jose, CA). We analyzed results with FlowJo software version 8.8.6 and 9.2 (TreeStar, Ashland, OR) to assess the purity of the leukocyte populations.

### cDNA-PCR and Pooling Strategy

We isolated RNA from each cell population using either the Allprep DNA/RNA Mini Kit or RNeasy Mini Kit (Qiagen, Germantown, MD). We synthesized cDNA from total cellular RNAs using a Superscript III First-Strand Synthesis System (Invitrogen, Carlsbad, CA). We created primary cDNA-PCR amplicons spanning 190 bp of exon 2 of macaque class I sequences with high-fidelity Phusion polymerase (New England Biolabs, Ipswich, MA as described previously [22]. For the cynomolgus macaques, each PCR primer contained one of 12 distinct 10-bp MID tags along with adaptor sequences for Roche/454 sequencing. After purification, we normalized primary amplicons to equimolar concentrations and pooled groups of 12 amplified products for GS FLX analysis. For the rhesus macaques each PCR primer contained one of 32 distinct 10-bp MID tags along with adaptor sequences for Roche/454 Titanium sequencing. After purification, we normalized primary amplicons to equimolar concentrations and pooled groups of 32 amplified products for GS Junior analysis. For the humans, each PCR primer contained one of 16 distinct 10-bp MID tags along with adaptor sequences for Roche/454 sequencing. After purification we normalized primary amplicons to equimolar concentrations and pooled groups of 16 amplified products for GS Junior analysis [24]. Each cell population was amplified with two different MID tags to minimize the effects of primer bias.

### Emulsion PCR and Pyrosequencing

For the cynomolgus macaque sequencing, we performed the emulsion PCR and pyrosequencing steps with GS FLX instruments (Roche/454 Life Sciences, Branford, CT) using standard chemistry according to the manufacturer's specifications (454 Life Sciences); the work was performed at the University of Illinois at Urbana-Champaign High-Throughput Sequencing Center and Northwestern University Division of Infectious Disease. We sequenced each amplicon pool of twelve products in one-sixteenth of a 70 × 75 mm Standard PicoTiterPlate (Roche/454 Life Sciences).

We performed the rhesus macaque and human sequencing, emulsion PCR and pyrosequencing steps with the GS Junior instrument (Roche/454 Life Sciences) using Titanium chemistry according to the manufacturer's specifications. We sequenced this amplicon pool of 32 and 16 products in two separate GS Junior 21 × 45 mm PicoTiterPlates (Roche/454 Life Sciences).

### Data Analysis

After image processing and base calling with GS FLX software (454 Life Sciences), we binned high-quality sequence reads by their respective MID tags and assembled the reads into contigs with 100% identity for each macaque using SeqMan Pro Version 8.0.2 (DNASTAR). We performed BLASTN or mosaik http://bioinformatics.bc.edu/marthlab/Mosaik analyses for the resulting contigs against a custom in-house database of macaque or human MHC class I sequences. Then, we compared transcript abundance levels between individual subsets in macaques. For normalization of each subset for statistical analysis, we divided the combined number of sequence reads for each distinct class I sequence from both MID tags by the total combined number of sequence reads for both MID tags. MHC class I nomenclature has recently been revised to reflect similar allelic lineages between closely related macaque species [25].

### Secreted MHC Production

To produce secreted MHC molecules, α-chain cDNAs of Mafa-B*134:02, were modified at the 3' end by PCR mutagenesis to delete exons 5-7 encoding the transmembrane and cytoplasmic domains and to add a thirty base-pair tail encoding the 10 amino acid rat very low density lipoprotein receptor (VLDLr), SVVSTDDDLA, for purification purposes [26]. sMHC-VLDLr were cloned into the mammalian expression vector pcDNA3.1(-) Geneticin (Invitrogen) and then sequenced to insure fidelity of each clone.

721.221 cells were transfected with sMHC Mafa-B*134:02TVLDLr by electroporation. After 48 hours incubation, cells were plated in 96 well plates (Falcon) in RPMI 1640 containing antibiotic. Transfectants were tested for production of sMHC molecules by a VLDLr specific ELISA [27]. Positive wells were subcloned into 96 well plates (Falcon) by limiting dilution. Individual wells with clonal cell populations were tested for the production of sMHC and high producers were expanded for inoculation into bioreactors in an AccuSyst-Maximizer (Biovest International, Minneapolis, MN).

### Peptide Purification

Approximately 25 mg of Mafa B*134:02TVLDLr molecules from the 721.221 cell line were purified over an affinity column composed of anti-VLDLr antibody (ATCC clone CRL-2197) coupled to CNBr activated Sepharose 4B (GE Healthcare, Piscataway, NJ). sMHC molecules were then eluted in 0.2 N acetic acid, brought up to 10% acetic acid, and heated to 78°C for 10 min. Peptides were separated from heavy and light chains by ultrafiltration in a stirred cell with a 3kDa molecular weight cutoff cellulose membrane (Millipore, Bedford, MA). The peptide batch was flash frozen and lyophilized. The peptides were then reconstituted in 10% acetic acid.

### Determination of Peptide Binding Motif

Following isolation, 10% of the peptide pool was subjected to 14 rounds of N-terminal sequencing by Edman degradation. A motif was generated by calculating the fold increase of each amino acid over the prior round. A hierarchy was determined based on the amino acid composition at each position [28].

### Reverse Phase HPLC

Peptides were reverse phase HPLC fractionated using a Jupiter Proteo C12 column (Phenomenex, Torrance, CA) on a Paradigm MG4 system (Michrom Bioresources, Auburn, CA). A standard CH_3_CN gradient was employed to generate approximately 40 peptide containing fractions. UV absorption was monitored at 215 nm.

### Mass Spectrometric Analysis

Peptide fractions were concentrated to dryness and reconstituted in 20 μl of nanospray buffer composed of 50% Methanol, 50% H_2_0, and 0.5% Acetic Acid. Nano-electrospray capillaries (Proxeon, Denmark) were loaded with 1 μl of each peptide fraction and infused at 1100 V on a Q-Star Elite quadrupole mass spectrometer with a TOF (time of flight) detector (Applied Biosystems, Foster City, CA). Ion maps were generated for each fraction in a mass range of 300-1200 amu. Independent Data Acquisition was used to select ions for fragmentation by tandem mass spectrometry (MS/MS). An amino acid sequence was assigned using the publicly available, web based MASCOT (Matrix Science Ltd., London, UK) and/or de novo sequencing.

### Fluorescent Peptide Staining

Fluorescent peptides were synthesized by Dr. Gary Case at the University of Wisconsin Biotechnology Center and by ProImmune Ltd. (Bradenton, FL). Fluorescein molecules were conjugated to lysine residues within each synthesized peptide. Two versions of each peptide with fluorescein conjugated lysine residues at two separate positions excluding the anchor residues were tested for each peptide. The peptides used in these experiments were Mafa-B*134:02 restricted NH_2_-GVFGFP(K-fluor)GR-COOH (fGR9), non-specific peptide NH2-YR(K-fluor)WIQLGL-COOH, Mafa-B*075:01 restricted NH_2_-ALPE(K-fluor)FTEL-COOH (fAL9), and Mafa-A1*063:01 NH_2_-SPIR(K-fluor)LPHW-COOH (fSW9).

Cells were aliquoted and then peptide was added at a final concentration of 100 nM. After 24 hours at 4°C cells were stained with CD20 PE, CD4 PerCP, CD3 PE Cy7, CD8 Pacific Blue, W6/32 APC, and CD14 A700. After 30 additional minutes of incubation at 4°C, the cells were washed twice and fixed with 2% PFA. Relative expression was calculated using the following formula (MFI ×/MFI No Peptide) * 100 where × was fGR9, fAL9, or fSW9.

### Statistics

In our sequencing analysis, we compared the frequency of an allele across different cell types by a chi-square test. We compared relative protein expression between select subsets in the M3 homozygous animals by a repeated measures ANOVA and paired student's T test using GraphPad Prism, version 5.0a for Macintosh (GraphPad Software, San Diego, CA http://www.graphpad.com). We used an unpaired student's T test to compare expression of Mafa-A1*063:01 across the different haplotypes in CD3+ cells.

## Results

### MHC transcription in human leukocyte subsets

We used Ficoll density centrifugation to isolate PBMC from the blood of two humans. Next, using magnetic separations, we isolated several distinct leukocyte populations based on the presence of well-characterized surface markers. We isolated CD4+, CD14+ and CD20+ cells to generally greater than 90% purity within the lymphocyte gate (data not shown). We isolated RNA from each separated subpopulation in addition to whole PBMC. Next, we performed cDNA synthesis and used universal MID-tagged primers to amplify a 581 bp fragment of all MHC class I genes. Every subset was subjected to two independent PCR amplifications with two unique MID tags. We then prepared these samples for pyrosequencing as previously described [22,24]. Finally we compared the sequences against an HLA reference database.

**Figure 2:**
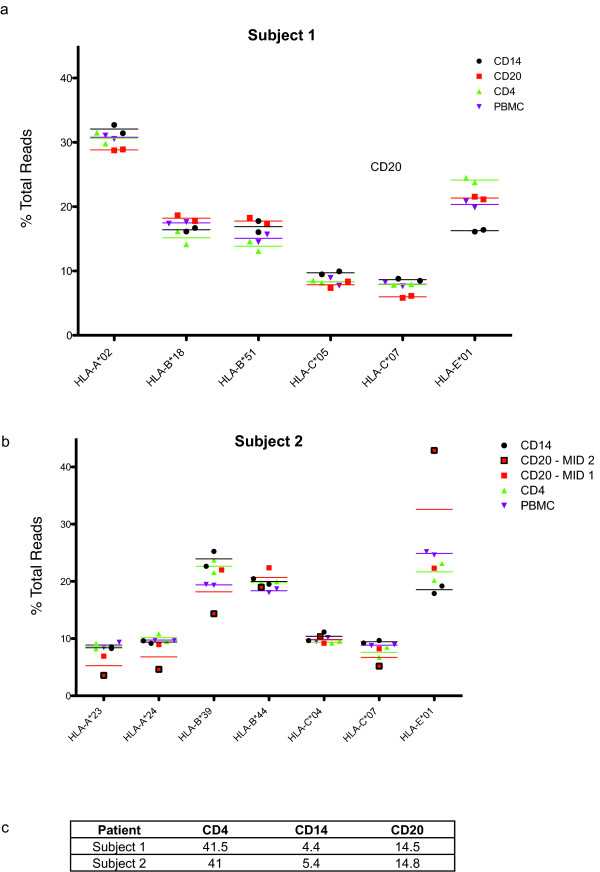
**Transcription of HLA in leukocyte subsets from two humans**. We normalized sequence reads for each allele within each leukocyte subset by dividing the number of reads from each class I sequence by the total number of reads identified from that subset. Plots show the mean and points represent independent PCR reactions using distinct MID tags sequenced in a single run. Alleles were distinguished at the two-digit level. a) Normalized transcript levels for the first subject. b) Normalized transcript levels for the second subject. Plots show both data points and the mean. c) Percentage that each subset contributes to the whole PBMC. For CD20 cells, data points from the PCR reaction with MID 1 are shown with red squares while those from MID 2 are shown with red squares and a black outline. A chi-square test was used to compare gene transcription between subsets for each cDNA species.

We created transcript profiles for each leukocyte subset based on the percentage of total transcripts each unique sequence constituted. We obtained an average of 3381 sequence reads per leukocyte subset. Next, we examined transcription of each allele across the four leukocyte subsets. There were small but significant differences in HLA transcription amongst lymphocyte subsets (p < 0.001) except for HLA-C*04 in the second individual (Figure 2). However, between the two individuals there were not consistent differences in transcription amongst different subsets. For example, HLA-B alleles were not consistently expressed at higher levels in any single subset. CD14 cells appear to express lower levels of HLA-E than the other cell types, but measurements in the second individual were variable. In particular, the levels of HLA-E detected in the second individual were not consistent between the two MID tags used for CD20 cells. The higher level of HLA-E detected using one MID tag in the CD20 cells led to generally lower detectable levels of the other HLA class I genes. The results demonstrate that the analysis is semi-quantitative and that we cannot determine whether one gene is transcribed at higher levels or if other genes are transcribed at lower levels. The first individual appears homozygous for HLA-A alleles with our current sequence coverage. Using our novel technique for examining MHC transcription, we were able to confirm previous results demonstrating that HLA-B is transcribed at higher levels in PBMC [16]. We also examined the contribution of each subset to the total PBMC (Figure 2c). We found that the percentage of each subset in total PBMC was consistent between the two individuals. These results do not discriminate between increased transcription of one gene or decreased transcription of the other MHC genes, therefore this method measures relative transcript levels and not absolute transcript levels. Nevertheless, these data confirm that our technique yields results qualitatively similar to those provided by qPCR.

### Differential mRNA Transcription of Mafa-B*134:02 in MHC-defined macaques

Next we examined transcription of MHC class I cDNA species in macaques which can express three-fold more distinct MHC class I transcripts than humans. We have previously described methods for rapidly genotyping MCM for their MHC haplotypes by microsatellite analysis and we have characterized all major MHC class I and class II alleles associated with these haplotypes [21-23,25]. We identified animals that included homozygotes and heterozygotes for MHC class I and class II alleles (Figure 1). These animals contained the M1, M2, M3, M4 and M7 haplotypes. Using MCM eliminated potential issues arising from uncharacterized animal genotypes and allowed us to study animals with defined degrees of MHC matching, thus simplifying the analysis.

We used Ficoll-Hypaque density centrifugation to isolate PBMC from macaque blood. Next, using magnetic separations, we isolated several distinct leukocyte populations based on the presence of well-characterized surface markers. We isolated CD4+, CD8+, CD14+, CD16+, and CD20+ cells to genearally greater than 90% purity within the lymphocyte gate (data not shown). Again, we isolated RNA from whole PBMC in addition to each separated subpopulation. Next, we performed cDNA synthesis and used universal primers to amplify a short, polymorphic 190 bp region of all MHC class I genes. Every subset was subjected to two independent PCR amplifications with two unique MID tags. We then prepared these samples for pyrosequencing as previously described [22]. Finally we compared the sequences against an MCM MHC class I BLAST database. We found all expected and previously discovered transcripts in the macaques' PBMC [22,25].

**Figure 3:**
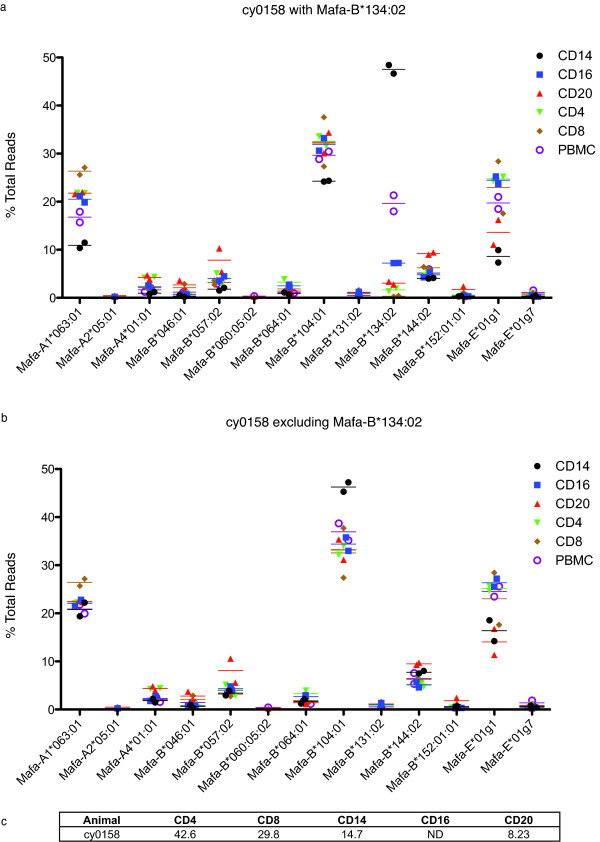
Differential transcription of Mafa-B*134:02 in animal cy0158. We normalized sequence reads for each allele within each leukocyte subset by dividing the number of reads from each class I sequence by the total number of reads identified from that subset. As before, plots show the mean, and each point represents the results of an independent PCR reaction with unique MID tag. a) Normalized transcript levels for cy0158 in each subset. b) Normalized transcript levels for cy0158 after removing the contribution from Mafa-B*134:02 c) Percentage that each subset contributes to the whole PBMC.

We created transcript profiles for each leukocyte subset based on the percentage of total transcripts each unique sequence constituted (Figure 3a). On average we identified 1342 reads per subset. We then compared MHC profiles between different subsets and noted that, while the profiles were similar between CD8+, CD16+, CD20+ and CD4+ cells, there was a clear difference between these populations and the CD14+ cells and PBMC (Figure 3a). Surprisingly, we observed that Mafa-B*134:02 transcripts were dramatically over-represented in the CD14+ cells compared to the other subpopulations (Figure 3a). In contrast, Mafa-B*134:02 transcripts were nearly undetectable in the CD8+ cells (Figure 3a). Again, we examined the contribution of each subset to the total PBMC (Figure 3c). The Mafa-B*134:02 transcripts detected in whole PBMC are consistent with the contribution of monocytes to this heterogeneous population. The CD16+ cells also had intermediate levels of CD14+ transcription which may explain the contribution of Mafa-B*134:02 to this cell subset. We found that transcription of Mafa-B*134:02 was significantly different between subsets (p < 0.0001). We repeated this experiment with cells from the same animal as well as with an independent M1/M1 animal and found the results to be consistent among experiments (data not shown).

### Consistent transcription of other M1 alleles

The extremely high transcription of Mafa-B*134:02 in the CD14+ cells made it difficult to assess the transcription pattern of other alleles in this leukocyte subset. To eliminate the effect of Mafa-B*134:02 transcription on the rest of our analysis, we renormalized the transcript percentages after eliminating the Mafa-B*134:02 reads (Figure 3b). This analysis, which excluded the contribution of Mafa-B*134:02 to each sample, demonstrated that the profiles were remarkably similar among subsets in the absence of Mafa-B*134:02. Although Mafa-B*104:01:01 was significantly elevated in the CD14+ cells (p < 0.0001), this difference was considerably less than that seen with Mafa-B*134:02.

### Differential MHC class I allele transcription in animals with non-M1 haplotypes

**Figure 4:**
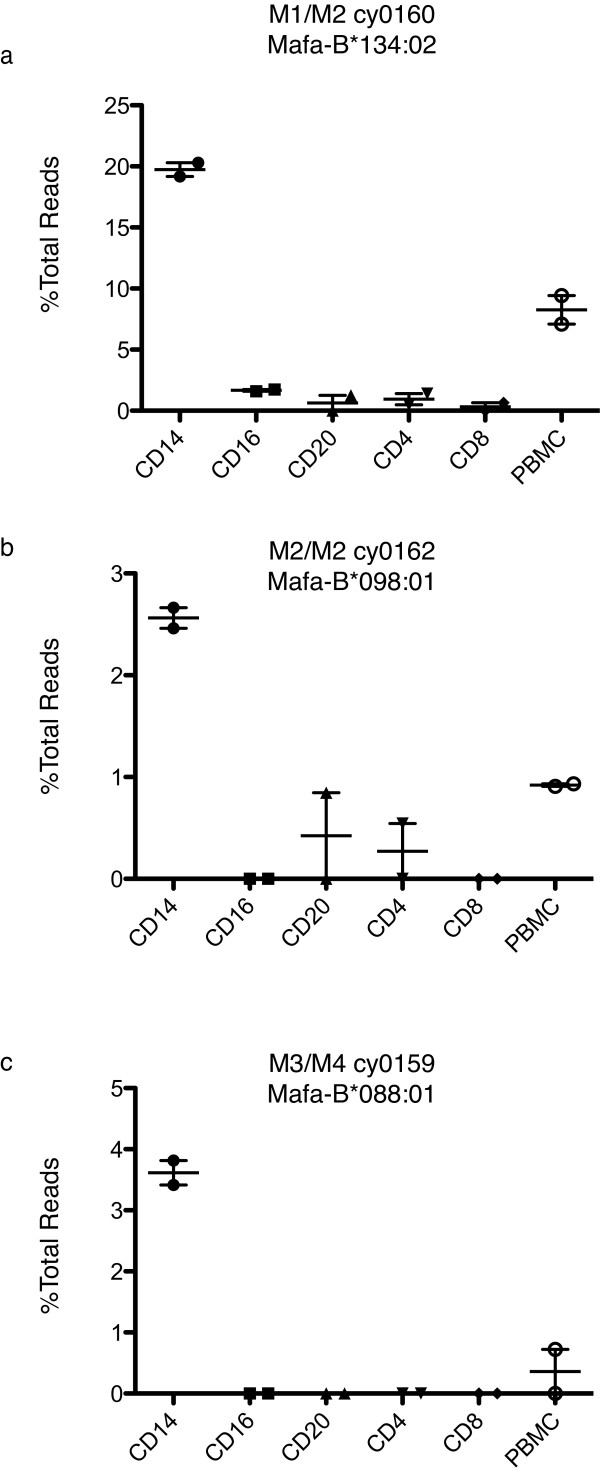
**Differential transcription of alleles in animals with different MHC haplotypes**. We normalized sequence reads for each allele within each leukocyte subset by dividing the number of reads from each class I sequence by the total number of reads identified from that subset. Points represent independent PCR reactions using distinct MID tags sequenced in a single run.. a) Expression of Mafa-B*134:02 in each leukocyte subset of M1/M2 animal cy0160. b) Expression of Mafa-B*098:01 in homozygous M2/M2 animal cy0162. c) Expression of Mafa-B*088:01 in M3/M4 heterozygous animal cy0159. Plots show standard error of the mean.

Next we assessed transcription of Mafa-B*134:02 in an animal that is heterozygous for the M1/M2 haplotypes. We found that the pattern in Mafa-B*134:02 transcription remained unchanged (Figure 4a). CD14+ cells continued to transcribe Mafa-B*134:02 at significantly higher levels than any other cell subsets (p < 0.0001). As expected, this heterozygous animal had reduced levels of Mafa-B*134:02 transcripts in CD14+ cells compared to the homozygous M1/M1 animals we analyzed. This suggests that there is a gene dosage effect in MHC class I transcription of this differentially expressed allele.

Next we examined MHC class I allele transcription in animals with different MHC haplotypes. In the M2/M2 background we observed that there was indeed another allele that was more highly transcribed in CD14+ cells (Figure 4b). We found that Mafa-B*098:01:01 was transcribed preferentially in the CD14+ cells (p < 0.0001). In the M2/M2 animal we observed lower overall transcription of this gene compared to other transcripts suggesting that it may be a minor MHC class I allele. However, it is important to remember that our measure of transcription is semi-quantitative; primers could be biased for certain cDNAs which ultimately biases the dominance hierarchy. Nevertheless, the relative transcription pattern of Mafa-B*098:01:01 was similar to that for Mafa-B*134:02. These differences were also detected in the M1/M2 heterozygous animal cy0160 (data not shown). However, in this animal, the level of Mafa-B*98:01:01 transcription was near our limit of detection.

In a final animal heterozygous for the M3/M4 haplotypes, we identified an RNA transcript on the M4 haplotype that also follows this variable transcription pattern (Figure 4c). In this animal, Mafa-B*088:01 was transcribed at higher levels in CD14+ cells (p < 0.0001). However, in this animal we did not identify an RNA species that is differentially transcribed from the M3 haplotype. We did not analyze either of these haplotypes in a homozygous macaque which may explain the low level of Mafa-B*088:01 transcription and lack of differentially transcribed alleles detected on the M3 haplotype.

### Differential transcription of Mamu-B*072 g and Mamu-B*098 g in the Indian rhesus macaque

Next we explored the transcription of classical MHC class I alleles in Indian rhesus macaques to assess whether differential transcription was conserved in a closely related macaque species. Three animals expressed Mamu-B*008 while the fourth animal expressed Mamu-B*017; both of these Mamu-B sequences are of special interested because they are associated with exceptional control of SIV replication. From these four animals we isolated three separate populations CD4+, CD14+, and CD20+ cells. We isolated RNA from each of these subsets in addition to unseparated PBMC and synthesized cDNA. Next we amplified the same variable region of the MHC class I gene as in the experiments with the MCM. Every subset was subjected to two independent PCR amplifications with two unique MID tags. We used the Roche/454 GS Junior to sequence these short products and obtained an average of 498 reads per cell subpopulation.

**Figure 5:**
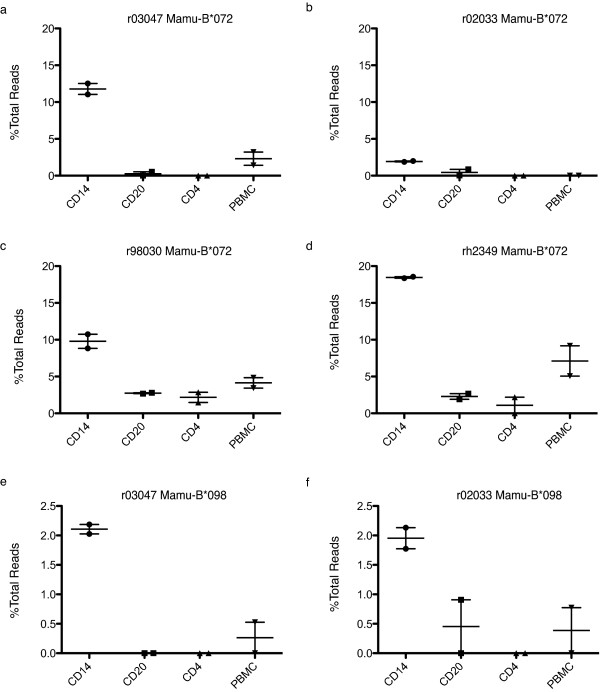
**Differential transcription of Indian rhesus macaque alleles**. We normalized sequence reads for each allele within each leukocyte subset by dividing the number of reads from each class I sequence by the total number of reads identified from that subset. Points represent independent PCR reactions using distinct MID tags sequenced in a single run. Alleles were distinguished at the two-digit level. Normalized expression of alleles in the Mamu-B*072 lineage across different subsets in animals a) r03047 b) r02033, c) r98030, and d) rh2349. Normalized expression of alleles in the Mamu-B*098 lineage across different subsets in animals e) r03047 and f) r02033. Plots show standard error of the mean.

While we did not find that Mamu-B*008 or Mamu-B*017 were differentially transcribed, we found that, similar to our results in the MCM, Indian rhesus macaques also have RNA species that are dominantly transcribed in the CD14+ cells. All four animals expressed Mamu-B*072 g at higher levels in the CD14+ cells (Figure 5a-d). The level of transcription amongst these animals varied, but the pattern of transcription within each animal was similar. We also identified a transcript present in two animals, Mamu-B*098 g, that is also dominantly transcribed in the CD14+ cells (Figure 5e-f). In the whole PBMC, Mamu-B*098 g borders on the limit of detection and would potentially be undetectable if we did not specifically examine transcription in CD14+ cells. In sum, these results indicate that differential transcription is conserved across at least two macaque species, suggesting it may have a biological role in these animals.

### Sequence motif identifies differentially expressed alleles

Next we sought to determine if these differentially expressed transcripts had similarities in their sequences that could explain the differential transcription. We aligned the differentially expressed rhesus and cynomolgus macaque sequences against all rhesus and cynomolgus macaque MHC class I B full-length sequences. We identified a short sequence motif in exon 3 encoding the alpha 2 domain that was only present in these differentially expressed RNAs (Table 1). We noted two synonymous differences and two nonsynonymous differences in codon 162 that resulted in a methionine to glutamic acid substitution in the predicted gene products. While we did not detect transcription of Mafa-B*098:05 in the M3 heterozygous animal, this cDNA species also shares this sequence motif. Morover, every MCM haplotype contains a transcript that encodes this motif, but we were unable to examine expression from a homozygous animal of each haplotype. Therefore, while the transcripts that that are differentially regulated appear to share this motif, it is unclear whether every transcript with this motif will be transcribed similar to the genes we identified here. Approximately four percent of all macaque MHC class I B alleles contain this sequence motif (Data not shown). This motif may play a role in the regulation of gene expression or may demonstrate that these RNA species evolved from the same ancestral class I gene. Importantly, Mamu-B*072 is encoded by the Mamu-h2-B02 locus as previously described by *Daza-Vamenta et al*. which is similar to other classical MHC class I B genes [1].

**Table 1:** Sequence motif unique to CD14 specific class I alleles

Class I B Consensus	G	C	C	G	C	G	G	A	C	A	T	G
CD14-Specific Expression Consensus	•	•	T	•	•	•	•	•	T	G	A	•

Class I B Translation	Ala			Ala			Asp			Met		

CD14-Specific Expression Translation	•			•			•			Glu		

All known full-length rhesus and cynomolgus macaque alleles were aligned. A motif specific to the alleles transcribed highly in CD14+ cells was identified. These CD14+ specific class I sequences were compared to the consensus sequence of all cynomolgus and rhesus MHC class I B alleles.

### Differential protein expression of Mafa-B*134:02

**Figure 6:**
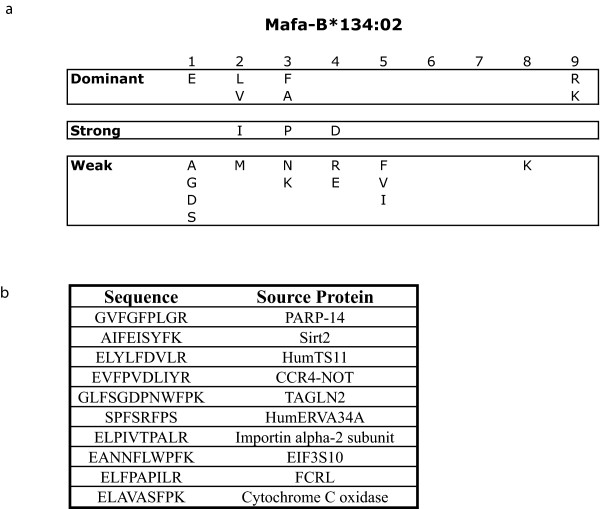
Binding motif defined for Mafa-B*134:02. a) Based on Edman degradation, residues were classified as dominant if they demonstrated a ≥ 3.5-fold increase in picomoles over the previous round of sequencing, strong if they exhibited a 2.5 to 3.5-fold increase and weak if they exhibited a 2.0 to 2.5 fold increase. b) Endogenous Mafa-B*134:02 ligand sequences identified by mass spectrometry.

Although we demonstrated that certain MHC class I sequences were regulated differentially at the transcript level, it was not clear whether this differential transcription lead to differences in protein expression. Previous studies have demonstrated that gene dose alone does not explain MHC class I allele surface expression in mice that are the heterozygous progeny of homozygous parents [17]. We optimized the use of fluorescein-conjugated peptides to assess the protein expression of alleles with matching peptide motifs. We have previously defined the binding motif for Mafa-A1*063:01 and Mafa-B*075:01, and we defined the binding motif of Mafa-B*134:02 using the same techniques (Figure 6a) [19]. We identified several endogenous ligands of Mafa-B*134:02 during this process (Figure 6b). We then chose several peptides with high binding affinity and replaced a non-anchor residue with a fluorescein-conjugated lysine. We tested two of these peptides and chose the peptide that bound with greater affinity. We used the fluorescein conjugated Mafa-B*134:02 specific peptide GR9 (fGR9) to assess protein expression of this MHC class I allele and then followed the same procedure for Mafa-A1*063:01 (fSW9) and Mafa-B*075:01 (fAL9).

**Figure 7:**
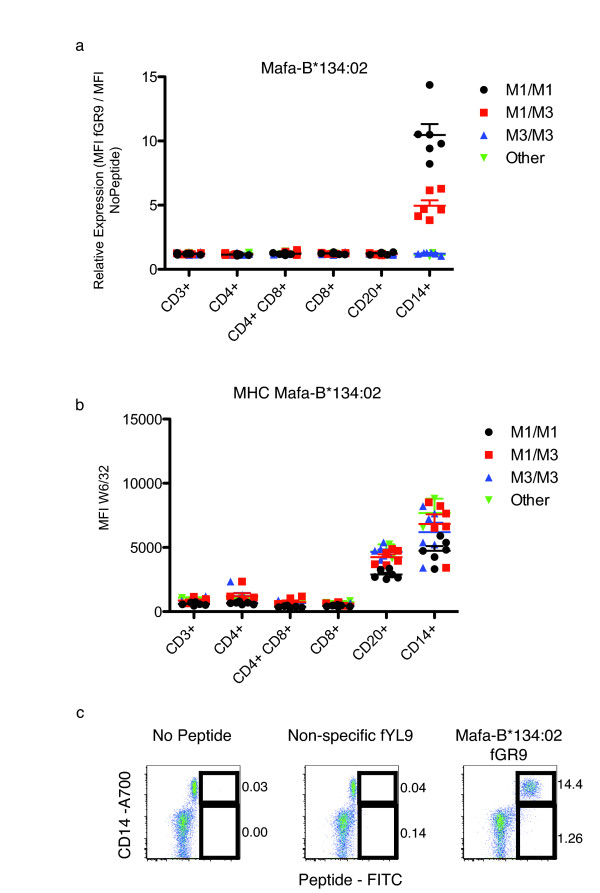
Measuring protein expression of Mafa-B*134:02 by fluorescent peptide. a) A fluorescent peptide, fGR9, was used to measure Mafa-B*134:02 expression in the PBMC. We looked at five different leukocyte subsets in 20 different animals. We determined relative expression using the following formula: (MFI fGR9)/(MFI No Peptide). b) MFI of W6/32 APC binding for each sample. c) Dot plots showing leukocytes where CD14 is plotted on the y-axis and peptide-FITC is plotted on the x-axis. Numbers to the right of the gates show the percentage of cells within the gate. Plots show standard error of the mean.

We found that fGR9 was specific for Mafa-B*134:02 and did not bind several other MHC class I alleles (data not shown). Without testing a comprehensive panel of single MHC class I expressing transfectants, it is impossible to formally eliminate the possibility of fGR9 cross reactivity with all Mafa class I alleles. We isolated PBMC from twenty animals (Figure 2b). Six M3/M3 animals, one M4/M7 and one M7/M7 lacked the Mafa-B*134:02 allele, six animals were homozygous for the M1 haplotype containing Mafa-B*134:02, and six were M1/M3 heterozygous animals. We normalized the level of peptide binding by the level of MHC expression in each leukocyte subset and then examined the percentage change compared to the no peptide controls. We found that when staining whole PBMC with fGR9, only CD14+ cells from Mafa-B*134:02+ animals stained positive (Figure 7a). Animals that are homozygous for Mafa-B*134:02 expressed the highest levels of this protein followed by the heterozygous animals. Levels of total MHC expression were also highest in the CD14+ cells although there were not large differences between the different animals. Additionally it appears that all CD14+ cells express Mafa-B*134:02 (Figure 7c). Expression of this allele does not appear specific to a particular CD14+ subset. These results indicate that the differences we detected in transcript levels correlated with differences in protein expression of Mafa-B*134:02.

**Figure 8:**
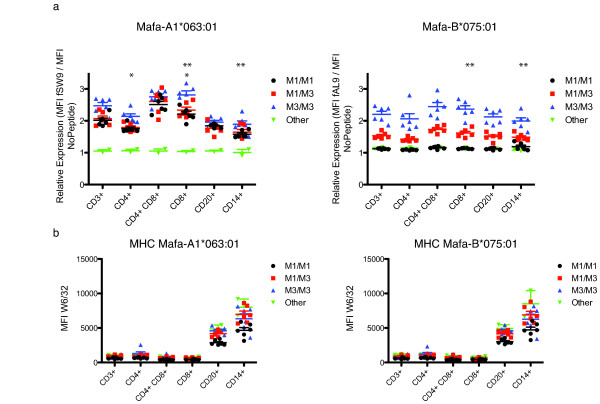
Measuring protein expression of Mafa-B*075:01 and Mafa-A1*063:01 by fluorescent peptide. Mafa-B*075:01 fAL9, Mafa-A1*063:01 fSW9 and non-specific peptide fYL9 were used to examine protein expression in six leukocyte subsets in the PBMC. a) Relative expression was calculated as previously described. A student's T-test was used to analyze the differences in MHC expression between select leukocyte subsets in the M3/M3 animals. The * symbols above each column indicate which columns were compared. (* p = 0.002; ** p = 0.009) b) Measuring total MHC class I expression by MFI of W6/32 APC binding for each subpopulation. The * symbols above each column indicate which columns were compared. (* p = 0.01) Plots show standard error of the mean.

We performed analogous expression assays for two MHC alleles, Mafa-A1*063:01 which is present on the M1, M2, and M3 haplotypes and Mafa-B*075:01 which is present on the M3 haplotype. These two alleles are both known to restrict SIV-specific CD8+ T cell responses. We focused our statistical analysis on the M3/M3 animals which appeared to express the highest levels of these MHC class I proteins. Interestingly, we found that expression of Mafa-B*075:01 (p = 0.01) and Mafa-A1*063:01 (p = 0.0009) was lower in CD14+ cells than CD8+ cells (Figure 8). These results suggest high levels of Mafa-B*134:02 are expressed in monocytes while other alleles are expressed at reduced levels in this cell type.

We also identified differential expression of Mafa-A1*063 between leukocyte subsets. We found that CD8+ T cells in M3/M3 animals express higher levels of Mafa-A1*063:01 (p = 0.002) compared to CD4+ cells, demonstrating that expression of several MHC proteins varies by leukocyte cell types. Moreover, Mafa-A1*063:01, which is present on both the M1 and M3 haplotype was expressed at a higher level on M3/M3 CD3+ cells compared to the M1/M1 (p = 0.004) and M1/M3 (p = 0.01) CD3+ cells. It is possible that differences in the leader peptide sequence between the Mafa-A1*063 transcripts present on the M1 and M3 haplotypes may account for these expression differences; however, we cannot eliminate the possibility that the fluorescent peptide is cross-reacting with another MHC class I allele on the M3. However, these results show that MHC expression varies dramatically by cell type and haplotype.

## Discussion

Cancer researchers first began describing the MHC class I loci over 50 years ago as they propagated tumors by transplanting them between mice [29]. Examining expression of particular alleles has classically been difficult due to the high degree of similarity among MHC sequences. Systems using artificial expression are typically utilized to assess how alleles are expressed but neglect how one allele can affect the expression of other alleles and, thus, are removed from *in vivo *studies. We assessed relative MHC class I transcript levels using a recently described Roche/454 pyrosequencing approach [22]. In humans, these studies identified greater transcription of HLA-B in PBMC than HLA-A or HLA-C [16]. However, they did not show differences in expression between the distinct leukocyte subsets. In contrast, examinations of macaque MHC expression revealed different levels of several MHC class I transcripts in distinct leukocyte subsets of both MCM and rhesus macaques. Furthermore, using a novel technique, we confirmed that these differences in transcript abundance translated to differences in protein expression of these gene products.

We found that contrary to the paradigm of universal expression of classic MHC class I alleles across different cell types, macaque leukocyte subpopulations exhibited differential expression of a subset of MHC class I sequences. We detected both increased transcript levels and increased protein expression of Mafa-B*134:02 in CD14+ cells when compared to other leukocyte subsets. Moreover, the only cells that appeared to bind a fluorescently labeled peptide specific for the Mafa-B*134:02 protein were monocytes suggesting this class I sequence may have a unique function in these cells. It is important to note that we did not examine other myeloid lineage cells including dendritic cells and mature macrophages by 454 analysis. It is entirely possible that other, more refined subsets, may have comparable expression differences with this or other class I sequences.

We have not yet identified responses restricted by Mafa-B*134:02 and were interested in studying expression of alleles that we know restrict SIV-specific responses. When we examined protein expression of two additional class I sequences known to restrict SIV-specific CD8+ T cell responses using fluorescent peptides we found that these MHC proteins were also differentially expressed in distinct leukocyte subsets. CD8+ T cells appear to express higher levels of Mafa-A1*063:01 and Mafa-B*075:01 than CD4+ and CD14+ cells. CD14+ cells generally express lower levels of these proteins providing evidence that Mafa-B*134:02 is expressed at the expense of other MHC proteins. Additionaly, we have not yet determined the mechanism by which these alleles are differentially expressed, but recent research by *Kulkami et al*. found that HLA-C alleles are regulated by microRNAs [30].

It is not clear how these differences impact the immune response generated by these different cell types. One possiblilty is that this differential expression could variably mold the immune response with the power to reshape immunodominance hierarchies. Mouse influenza experiments have previously demonstrated that the degree of protein expression of MHC class I alleles can affect the magnitude of CD8+ T cell immune responses generated by the host [17]. A monocyte-tropic virus might generate different responses than a CD4 T cell-tropic virus expressing similar protein sequences in macaques.

This may have particular implications for SIV, the dominant experimental model for studying HIV pathogenesis. SIV is known to have various tropisms that shift throughout infection. SIVmac239 is a CCR5-tropic virus and initially infects effector CD4 T cells. These cells are massively depleted in the gut during the first weeks of infection [31]. As SIV continues replicating it switches tropism over time to begin infecting CD4 T cells with other memory phenotypes [6]. The virus is also capable of infecting macrophages, the tissue-resident, lineage descendants of CD14+ monocytes. It is unclear how the tropism of the virus might affect the CD8+ T cell responses generated within these individuals. Our results suggest that different viral targets may elicit different SIV-specific CD8+ T cells due to differences in MHC class I expression. This may also have a profound impact on vaccination and the immune responses generated depending on the cells targeted by the vaccine regimen.

Still, it is important to note the macaque MHC class I region differs from the human class I region in several ways. Intriguingly, macaques can express more than three times the number of class I transcripts as humans [1]. In addition to MHC diversity at the population level, there are several studies that suggest expressing more total alleles is advantageous in host pathogen interactions, a phenomenon known as the heterozygote advantage [32-41]. If having more MHC class I alleles is advantageous, it is important to understand why humans do not have additional MHC class I alleles on each chromosome. Some research suggests that having additional MHC class I alleles leads to loss of certain CD8+ T cells that are self-reactive, thus limiting the repertoire of T cells [42]. These issues are further confounded in light of macaques expressing a large number of MHC class I genes. The differential expression of MHC class I alleles that we observed in the macaque may represent an evolutionary adaptation to accommodate the large number of alleles expressed in these animals.

## Conclusions

Ultimately these results demonstrate that certain alleles in the macaque MHC class I are expressed differentially in distinct leukocyte subsets. Additionally, these differences are conserved across multiple haplotypes and species, suggesting there may be functional significance to this differential expression. Furthermore MHC class I known to restrict CD8+ T cell responses during SIV infection also appear to be differentially expressed providing evidence that this phenomenon is not unique to the alleles we identified by sequence analysis and may be a universal aspect of macaque MHC class I expression. We believe this demonstrates the first example of differential classical MHC class I expression in primates.

## Competing interests

The authors declare that they have no competing interests.

## Authorship Contributions

JMG wrote manuscript and performed experiments. RWW assisted in performing experiments and analyzing results. SML assisted in sequencing reactions and analyzing results. BNB developed informatics to analyze sequencing results. JAK assisted with sequencing and optimized sequencing protocols. BJB assisted with cell processing and separations. JJL assisted with cell processing and separations. OEH developed peptide binding motifs and assisted in writing. KJK provided assistance in sequencing. KWB assisted in statistical analysis. SMW provided sequencing facilities and assisted in sequencing. WHH oversaw development of peptide binding motifs. DHO conceived of experiments and assisted in writing. All authors read and approved the final manuscript.
